# Assessing service and treatment needs of young people who use illicit and non-medical prescription drugs living in Northern Ontario, Canada

**DOI:** 10.12688/f1000research.16464.2

**Published:** 2019-10-28

**Authors:** Thepikaa Varatharajan, Pamela Sabioni, Cayley Russell, Joanna Henderson, Benedikt Fischer, Sarah Miles, Jürgen Rehm

**Affiliations:** 1 Institute for Mental Health Policy Research, Centre for Addiction and Mental Health, Toronto, ON, Canada; 2 Department of Psychiatry, University of Toronto, Toronto, ON, Canada; 3 The Margaret and Wallace McCain Centre for Child, Youth and Family Mental Health, Centre for Addiction and Mental Health, Toronto, ON, Canada; 4 Centre for Criminology & Sociolegal Studies, University of Toronto, Toronto, ON, Canada; 5 Institute of Medical Science (IMS), University of Toronto, Toronto, ON, Canada; 6 Department of Psychiatry, Federal University of São Paulo, São Paulo, Brazil; 7 Department of Anesthesia and Pain Management, Toronto General Hospital, Toronto, ON, Canada; 8 Dalla Lana School of Public Health, University of Toronto, Toronto, ON, Canada; 9 Institut für Klinische Psychologie und Psychotherapie, Technische Universität Dresden, Dresden, Germany

**Keywords:** Canada, drug use, needs assessment, Northern Ontario, services, treatment, youth, young adults

## Abstract

**Background:** The use of illicit and prescription drugs for non-medical purposes among youth and young adults living in Northern Ontario communities is a major public health concern. This problem is amplified in that there is insufficient knowledge on the types of services and treatment centers available for and utilized by young people with substance use issues in Northern Ontario. This needs assessment study aims to examine the service and treatment needs of youth and young adults who use drugs in Northern Ontario communities.

**Methods/Design:** A mixed-methods study design will be used to assess the service and treatment needs of youth and young adults (aged 14-25) who have used one or more illicit drug (excluding cannabis) and/or psychoactive prescription drug for non-medical purposes for at least 3 months and on at least 10 days in the last month. Participants will be recruited from approximately ten Northern, remote and rural communities across Northern Ontario using a mobile research lab. Eligible study candidates from each community will be asked to partake in a focus group and questionnaire exploring service and treatment utilization and needs. We will additionally collect basic socio-demographic information as well as examine patterns of problematic drug use. Interviews with service providers and community organizers will also be conducted in each community.

**Discussion**: Findings from our study will highlight the availability, accessibility and utilization of existing services; identify the gaps and barriers in current service provision; and provide insight into the service and treatment needs of youth and young adults who use drugs in Northern Ontario communities. Assessing the needs of young people who use drugs will allow service providers, community organizers and health policymakers to improve addiction-related services and treatment centers in Northern Ontario.

## Introduction

The high prevalence of illicit drug (such as heroin, cocaine, and amphetamines) and non-medical prescription drug (such as oxycodone, barbiturates and anti-depressants) use has become a major global health concern, with the non-medical use of prescription opioids (POs) especially prevalent in North America ^1– 4^. According to the Canadian Tobacco, Alcohol and Drug Survey (2015), approximately 2% of Canadians in the past-year used at least one of the five following illicit drugs: cocaine or crack, ecstasy, speed or methamphetamines, hallucinogens or heroin ^5^. Moreover, approximately 60% of people who use illicit drugs are between the ages of 15 to 24, and this age group is more likely to use illicit drugs heavily, and in more risky ways ^6, 7^.

In Ontario, the most commonly used illicit drugs among young people after cannabis are cocaine, ecstasy and hallucinogens ^8^. The most frequently used prescription drugs for non-medical purposes among youth and young adults are opioid pain relievers, drugs prescribed for attention deficit hyperactivity disorder and tranquilizers ^5, 8^. In 2015, 21.5% of Ontario students from grades 7–12 reported using any drug other than cannabis in the last year, including 12% who used a prescription drug (e.g. Percodan, Tylenol #3, codeine) for non-medical purposes ^8^. Among young adults (18–29 years old), approximately 5% used POs for non-medical purposes in the past year ^9^.

Substance use patterns among youth and young adults living in Ontario vary with geographical setting (e.g.; northern vs. southern jurisdictions and rural vs. urban communities) ^10– 12^. These differences could be due to economic, cultural, social and educational factors ^11^. For example, opioid dependence among young people has become a widespread issue in Northern Ontario ^10, 13, 14^. Findings from a study by Kurdyak *et al.,* (2017) indicate that PO misuse is significantly higher among youth and young adults in Northern Ontario than the rest of the province ^15^. As an indicator, the rate of methadone maintenance treatment patients per 100,000 between the ages of 15 to 24 in 2014 was approximately 2-fold (118 per 100,000) and 6-fold (325 per 100,000) higher among the North East and North West Local Health Integrated Networks (LHINs), respectively, compared to the other LHINs in Ontario (50 per 100,000) ^15^.

Survey findings suggest that the past-year use rates of illicit and non-medical prescription drugs among youth and young adults living in the Northern LHINs (North West, North East and North Simcoe Muskoka) are generally similar to the rest of Ontario ^8, 9^. Approximately 13% and 11% of youth attending schools in the North East and North West LHINs respectively, used a non-prescription medical drug in the past year, while the provincial estimate was 12% ^8^. However, use rates in the Northern LHINs may be underestimated due to sampling procedures and survey inclusion and exclusion criteria (e.g. youth and young adults not attending schools; young people residing in hospitals, and prisons; First Nation communities; and transient populations such as the homeless or marginally housed are excluded) ^8– 10^.

In addition, Northern Ontario is home to a large number of Indigenous communities ^16, 17^. Indigenous youth are considered particularly vulnerable to illicit substance misuse and abuse due to the historical and systemic impacts of colonization, inter-generational violence/trauma and cultural genocide ^18^. Some First Nations youth begin using drugs, such as ecstasy and POs, at ages as young as nine years old ^19, 20^. In the Nishnawbe Aski Nation, which represents 49 First Nation communities within Northern Ontario with a total population of 45,000 people, up to 50% of the youth misuse POs (mainly Oxycontin) ^21, 22^. Furthermore, solvent abuse (especially inhaling gasoline) was found to be a major concern among First Nation and Inuit youth living in Northern Ontario communities ^10, 23, 24^.

Across Northern Ontario, there are a variety of community health and social services and treatment centers available to individuals with mental health and addiction issues ^10, 25– 27^. Approximately 34 and 44 of these mental health and addiction service providers are funded by the North West and North East LHINs, respectively ^17, 28^. However, it is unclear whether these services and treatment centers are being accessed and utilized by young people who use drugs. For example, in 2017 it was reported that children and youth (aged 0–24 years) living in the North West LHIN had the highest rates of emergency department visits (37.7 per 1000 population) for mental health and addiction problems compared to the rest of Ontario (16.3 per 1000 population) ^29, 30^. Although more children and youth are seeking help in these socially disadvantaged areas, the use of emergency departments for mental health and addiction problems may indicate a lack of timely access to primary care or community-based mental health and addictions services and treatment centers ^29, 30^. Furthermore, high rates of emergency department visits may also indicate a low awareness of community-based services in Northern Ontario communities ^29, 30^.

Moreover, there is currently limited knowledge with regards to how well the existing services and treatment centers in Northern Ontario are addressing the needs of young people with substance use issues. An addictions service needs assessment study conducted in Ontario-based First Nations communities - of which a majority were located in Northern Ontario - reported that the addiction-related education, promotion and prevention programs in their communities mainly targeted adults ^19^. For Indigenous youth and young adults who use drugs, there have been calls for culturally-based and culturally safe services, particularly those that are informed by the institutional and intergenerational trauma experienced by Canada’s First Nations, Métis and Inuit peoples ^19, 31^. Previous studies have also emphasized the importance of providing adequate support to transitional-aged youth (ages 18–24); Lesbian, Gay, Bisexual, Trans and Queer (LGBTQ)-competent services; and gender-specific services in Northern, rural and remote regions ^26, 27, 32, 33^. A more thorough understanding of the service and treatment needs of youth and young adults who use drugs in Northern Ontario is essential in identifying and addressing the gaps in current service provision and utilization.

## Methods/design

### Aims and objectives

This study aims to determine the service and treatment needs of youth and young adults (aged 14–25) who regularly use illicit and non-medical prescription drugs in communities across Northern Ontario. The specific objectives of this community-based needs assessment are to:

1. Examine the service and treatment needs of young people who use drugs in Northern Ontario.
2. Assess the availability, accessibility and utilization of existing services and treatment centers in Northern Ontario for youth and young adults with addiction and substance use issues.
3. Identify the gaps and key barriers to service and treatment utilization among young people who use drugs in Northern Ontario communities.

### Setting

This study will be conducted in approximately ten remote and rural communities across Northern Ontario, including: North Bay, Thunder Bay, Sudbury, Sault Ste. Marie, Timmins, Kenora, Fort Frances, Dryden, Smooth Rock Falls and Kapuskasing. Other communities might be added to the study according to availability and feasibility.

### Study design

This study will use a convergent parallel mixed-methods approach, which includes conducting: 1) a self-administered questionnaire and a focus group with young people between the ages of 14–25 years; and 2) one-on-one interviews with service providers who directly work with youth and young adults who use drugs in their communities ^34, 35^.

### Piloting of the procedures

To pilot the questionnaire and focus group questions and the study procedures in the mobile research lab, we contacted community organizers from addiction and substance use related services in the Greater Toronto Area and surrounding area to help with participant recruitment. Youth and young adults (aged 14–25) who use illicit and non-medical prescription drugs were eligible to participate and were compensated with a $20 gift card for their participation. The data collected from our pilot sessions will not be used for data analysis. At the end of the pilot study sessions, we asked participants to provide feedback on our focus group and questionnaire questions.

### Questionnaire and focus group methods with youth

***Participant recruitment.*** A collaborative approach will be used to recruit young participants, where study personnel will aim to network and build relationships with health and social service organizations within each community prior to entering the field. Key community collaborators will be asked to inform their colleagues, community partners, and young people about the study. Posters and handouts containing study details (such as date, time, contact information and location of data collection procedures) will be sent to community collaborators to distribute and post in their services. If possible, community collaborators will also organize focus groups (4–5 participants per group) in advance for study investigators.

Researchers will also attempt to directly recruit participants by informing individuals who approach the mobile research lab about the study. Participants who have completed the study will also be encouraged to ‘spread the word’ about the study to their peers using a respondent-driven sampling technique, which has been approved by the Centre for Addiction and Mental Health (CAMH) Research Ethics Board (REB) ^36^. Participants who would like to recommend the study to their peers will be given five coupons. For every eligible participant that is recruited, a $5.00 gift card will be given to the individual who made the recruit. An individual can earn up to a maximum of $25.00 for participant recruitment. Research staff will keep track of the coupons distributed by recording the coupon code under each participant’s unique study code.

***Study procedures.*** Two study personnel will be travelling to each of the communities, using the CAMH mobile research lab [designed for an earlier study: Wells *et al.*, 2011 ^37^]. The mobile research lab is a custom-built cargo van that allows researchers to conduct data collection procedures in communities across Northern Ontario, including remote and rural communities ^37^. The mobile lab will be parked in a location that is easily accessible and visible to participants in each community.

All study candidates will be screened for eligibility either in-person or over the phone by study staff using a screening form. The screening process should take less than 5 minutes. Eligible participants must meet the following criteria: a) be between 14 and 25 years of age at the time of consent; b) have used illicit psychotropic drugs (e.g., cocaine, ecstasy, inhalants) and/or prescription drugs for non-medical purposes, not including cannabis, for at least 3 months; and c) used one or more of these drugs on at least ten days within the past month. Including youth who are frequently misusing drugs (i.e. on a daily/more than weekly basis) will identify those individuals who are more likely to seek services and treatment for their drug use. Youth aged 14–16 years old will be eligible to participate as the risks associated with this study are low. Participants will be excluded if they are: a) unable to provide informed consent or b) unable to speak English. Eligible participants, who have been screened over the phone and have verbally agreed to participate in the study, will be asked to meet research personnel at a specified date/time/location to partake in research activities. A unique and confidential code will be assigned to participants to confirm their identity on the day of data collection. Eligible study candidates who were screened in-person will be asked to immediately partake in study activities.

All eligible participants who are interested in partaking in the study will be asked to sign a consent form ( Supplementary File 1). We will not seek parental consent for youth between the ages of 14 to 16 years old because they may be street-involved, disconnected from their parents, or not want to reveal their drug use to their parents. In compliance with Public Health Agency of Canada’s Research Ethics Board (REB) guidelines for seeking consent from individuals less than 16 years of age in Ontario, study staff will ensure that all participants understand the study and the possible consequences of participation by reviewing the consent form with participants and clarifying any questions participants may have. To ensure that no false decisions were taken with respect to the ability of participants to give informed consent, research staff will be trained by a CAMH clinical physician to judge capacity by assessing acute intoxication and other relevant impairments that may interfere with the participant’s ability to consent.

Once the consent forms have been signed, participants will be asked to partake in a 30-min, semi-structured focus group, which will be audio-recorded ( Supplementary File 2). In each focus group, there will be two moderators and 2–5 participants. If only one participant is available, a one-on-one interview will be conducted using the same questions in the focus group to capture the thoughts and perceptions of the participant. Both moderators will facilitate the discussion using open-ended questions and maintain an audit trail of emerging themes through memo writing and reflections during focus group sessions.

After completing the focus group session, participants will partake in a 20-min self-administered questionnaire ( Supplementary File 3). Depending on their preference, participants can choose to complete the questionnaire on paper or using a tablet (off-line Qualtrics Survey software) ^38^. Study staff will offer to administer the questionnaire verbally to accommodate participants who might have low literacy skills and/or prefer verbal completion. Both the focus group and questionnaires will explore the participants’: 1) drug use; 2) knowledge of and experience using the available services and treatment centers in their community for their drug use; 3) personal barriers to accessing and utilizing addiction-related services and treatment centers; and 4) service and treatment needs. At the end of the study session, participants will be compensated with a $20 gift card and will receive a listing of available addiction and mental health services and treatment programs in their community.

### Interview methods with service providers

***Participants and recruitment.*** In each community, we will recruit 3–5 service providers to participate in a semi-structured, one-on-one interview. Eligible participants will hold a formal position in the community in which they serve or interact with young people who use drugs (e.g., social worker, addictions counsellor), or hold a position in which they influence services, decisions or policies that affect young people who use drugs (e.g., police chief, town councilor).

***Study procedures.*** Interviews with service providers will either be conducted in-person while visiting each community or over the phone. Prior to starting the 30-minute interview, participants will provide written informed consent. The interview, which will be audio-recorded, will comprise of open-ended questions that will allow service providers to express themselves freely on the following topics: 1) their role in the community; 2) patterns of drug use among young people in their community; 3) the availability, accessibility and utilization of addiction-related services and treatment centers in their community and 4) the service and treatment needs of youth and young adults with addiction and substance use issues ( Supplementary File 4). Service providers will not be compensated for their participation as they will also be involved in the study in a professional capacity (i.e. assisting in participant recruitment and study logistics).

### Confidentiality and security of data

During the eligibility screening session, youth and young adults will be asked to provide their full name and telephone number. If the participant is eligible, they will receive a unique participation study code that cannot be associated with their personal information. To maintain confidentiality and anonymity, the eligibility screening forms will be kept separate from all other data collection materials and eligible study candidates will sign the consent forms using only their initials. At no point during the questionnaire or focus group sessions will we require a participant to report any personal identifiers (such as name, phone number, address, and date of birth). Service providers who agree to participate in the study will also be assigned a unique study code to protect their confidentiality and anonymity.

The unique study codes assigned will be used to link study data for the individual participant. Only members of the research team will have access to this code. Paper documents will be de-identified and stored in a locked filing cabinet in the CAMH mobile lab or in CAMH offices. Electronic files will be saved in encrypted devices in secure files that are password protected/locked. Hard copy files of data and electronic files will be held and destroyed according to usual research standards.

### Sample size

We anticipate recruiting at least 90 youth and young adults and 30 service providers to participate in this study. The total number of participants required for the qualitative portion of this study will be based on the principle of saturation sampling. We will continue to recruit participants until a sufficient saturation of responses or information (themes or categories) is achieved from the focus group and interviews.

For the quantitative portion of the study, we determined a sample size of at least N=90 for youth and young adults fulfilling the inclusion criteria. Such a minimal sample size would allow us to detect the following effect size for associations: a sample size of 90 achieves 80% power to detect a difference of 0.29 between the null hypothesis of zero and the alternative hypothesis correlation of 0.29 using a two-sided hypothesis test with a significance level of 0.05 ^39– 41^. As for mean differences of subgroups, group sample sizes of 45 and 45 achieve 80% power to reject the null hypothesis of zero effect size when the population effect size is 0.60, and the significance level (alpha) is 0.05, using a two-sided two-sample equal-variance t-test. Effect Size was defined as d = (μ1 - μ2) / σ. According to the classification of Cohen, this corresponds to sufficient power to detect medium effect sizes ^42– 45^.

### Data analysis plan

For the qualitative portion, audio-recorded data from the focus groups and interviews will be transcribed by research staff. A constant comparative method will be used to compare and contrast the data and an inductive qualitative thematic analysis approach will be used to identify key issues and themes regarding service use and needs ^46, 47^. Themes identified in the data will be coded into categories and then interpreted ^46, 48^. A qualitative computer software package, NVivo ^TM^ (version 11.0), will be used to store and organize the various codes derived from the data ^49, 50^.

For the quantitative portion of the study, a basic descriptive analysis will be conducted to assess the demographic characteristics and drug use patterns of young participants (e.g., age, gender, education level, income level, prevalence of drug use, etc.). A multivariable analysis using the statistical analysis software SAS (version 9.4) will be conducted to distinguish the relationship between characteristics (e.g. regression, analysis of variance) ^51^.

Data from the qualitative and quantitative portion will be merged, analyzed and interpreted to form final conclusions ^34^. To ensure that the needs and experiences of the youth and young adults are appropriately captured and reported, we will seek engagement with service providers, community organizers, individuals with lived experiences and First Nations, Inuit and Métis community partners and youth whenever possible to help analyze and interpret the data. Before finalizing all relevant reports and publications, all collaborators will have the opportunity to review research findings.

### Ethics

Study methods in this protocol have been reviewed and approved by the CAMH REB (protocol reference #133/2015). Ethics approval has also been granted by the Lake of the Woods District Hospital REB in Kenora and Sault Area Hospital REB in Sault Ste. Marie. Further ethics approval is not required.

### Dissemination of study outcomes

Each community partner will receive a final report with overall and community-specific study results. Study results will be published in a peer-reviewed journal and widely disseminated to service providers, agencies and systems relevant to Northern Ontario communities.

### Study status

We have completed data collection and are currently in the process of writing the results.

## Discussion

Findings from this study will add to our limited knowledge of the drug use patterns of youth and young adults across Northern Ontario. This study will also identify both the availability and utilization gaps for addiction and substance use related services and treatment centers for young people who use illicit and non-medical prescription drugs living in Northern Ontario communities. This study may also be valuable for future community-based research studies that intend to use a mobile research lab in Northern, remote and rural communities.

We anticipate that the results from this study will help service providers, community organizers and health policymakers make informed decisions on how to improve the current provision of addiction-related services for young people who use drugs across Northern Ontario communities. As a result, youth and young adults between the ages of 14 and 25 who have substance use problems may find that their needs are being addressed. The young individuals who participated in the study may find the list of community specific services and treatments centers provided to them at the end of the study to be a useful resource for their addiction and substance use issues. We also anticipate that research participants will benefit from the gift cards provided at the end of the study for participation and/or participant recruitment.

In this study, there are no inherent physical risks for participants (youth, young adults and service providers). Prior to starting data collection activities, we will let participants know that they may withdraw from the study at any time without prejudice or penalty and that the research staff will be willing to answer any questions or concerns that might arise during the study session. We will also remind participants that they always have the right to decline to answer any question that makes them feel uncomfortable or they find difficult to answer. Furthermore, study staff will inform all participants that all quotes used in this study will remain anonymous and will be reported in such a way that the identity of the participant is not revealed. Quantitative data will only be reported in aggregate.

For youth and young adults, there are no risks associated with completing the questionnaire. There may be some risks associated with partaking in the focus groups. For example, a participant may experience strong feelings about disclosing their drug use or a participant’s drug use could possibly be revealed by other fellow focus group participants. However, we believe these risks of disclosure are no greater than those caused by usual standard of care (e.g., attending addictions treatment or support group). In addition, we anticipate that since all focus group participants are using drugs, the youth will be less hesitant to share their experiences. To ensure the privacy of all participants, we will remind all focus group members to maintain confidentiality post research activities. We will also phrase focus group questions to elicit responses about drug use in the community, rather than about a participant’s own drug use.

There are a few limitations to this study. First, we believe that despite our efforts, fewer youth may participate in our study than anticipated. This could be due to multiple reasons including, but not limited to, lack of awareness of study, lack of interest, forget to attend study sessions, not enough motivation to complete data collection procedures, and/or fear of being stigmatized as a drug user by a family member or friend. Second, we anticipate that we will not be able to target as many youth and remote communities as planned if there is insufficient support from community providers and outreach workers. Third, the external validity of the results may be jeopardized due to self-selection bias. This is because participants may only volunteer to participate in the study to receive the gift card incentive ^52^. Fourth, we foresee both respondent and interview biases in this study. Richness of the questionnaire data may be limited as participants may skip answers or falsify their responses. This may be because participants are limited in time, have low literacy or feel uncomfortable answering questions about their drug use. Focus group participants may have poor memory, speak dishonestly, or alter their responses to fit in with the popular response or due to the presence of an opinionated or dominating participant. Finally, we anticipate that participant recruitment and data collection procedures may be hindered due to time and weather constraints ^36, 52, 53^.

A follow-up study assessing the specific service needs of Indigenous youth and young adults who use drugs in Northern Ontario is imperative for future research. This study should adopt a culturally-appropriate and culturally-informed collaborative approach and should attempt to reach rural and remote Northern Ontario communities, including the Treaty 9 First Nation reserves. Future needs assessment studies conducted in Northern Ontario should also focus on addressing the service gaps experienced by specific subgroups of youth (e.g., Francophone youth, homeless youth and LGBTQ youth) who use drugs.

## Data availability

No data is associated with this article.

## Supplementary material

Supplementary File 1: Youth consent form.

Supplementary File 2: Youth focus group guide.

Supplementary File 3: Youth questionnaire.

Supplementary File 4: Key informant interview guide.
