# Supplementary material for: Assessing service and treatment needs of young people who use illicit and non-medical prescription drugs living in Northern Ontario, Canada
Source: F1000Res. 2019 Oct 28;7:1644. Originally published 2018 Oct 16. [Version 2] doi: 10.12688/f1000research.16464.2 (PMC7025771; doi:10.12688/f1000research.16464.2)
Supplement: Supplementary file 1 [file f1000research-7-23219-s0000.tgz › 096925bb-9621-45cf-948e-2200efbea4bb_NODUS.Protocol.F1000Research.Youth.ConsentForm.docx]

­

**Survey and Focus Group Information Letter**

**NODUS Needs Assessment**

**Northern Ontario Drug Use and Services Needs Assessment**

*Principal Investigator: Dr. Pamela Sabioni*

We are inviting you to be a participant in a research study that involves a short survey and a focus group, which is a small group discussion. Study participants should regularly use drugs and be between 14 and 25 years old.

From this research, we hope to understand the characteristics of youth and young adults who use drugs in Northern Ontario, such as the types of drugs they use, their health, and their social characteristics. We also hope to learn about what services they use and what services they need. This study will help us to recommend services that could be added in Northern Ontario communities to help young people who use drugs.

STUDY APPROACH & METHODS

Your voluntarily participation will involve:

1. A survey (max 30 min). We will ask about your social characteristics, physical and mental health, drug use, and service use.
2. A focus group discussion that will last up to 1 hour. You and about 3-4 other young people who use drugs will tell us about drug use in your community, what services are available in your community, what services are not available, and how you choose what services to use. We will audio record the focus group.

We are doing the study in up to 10 communities across Northern Ontario. Approximately 120-250 young people will do the survey and focus group. We hope that 12-25 young people from your community will participate.

BENEFITS

Your participation will help us better understand what new services would be most helpful in communities across Northern Ontario and what kinds of things might give you better access to services in the future. If you would like, we can give you information about the services (such as harm reduction, substance use prevention, addictions treatment) that are already available in your community.

RISKS & DISCOMFORTS

Your participation in this study may cause you some emotional distress or inconvenience, including the time and effort it takes to participate in the survey and focus group. There is also a risk that somebody might find out that you use drugs, either because they know you did the study or because of what you say in the focus group. However, you do not have to discuss your own drug use in the focus group. We might ask you some personal questions (for example, survey questions about your physical or mental health problems, or involvement in activities that are risky to your health) that

Participant Initials: _____

you may find uncomfortable. If you need help with any issues that come up in the study, we will give you the contact information for services that can help you.

COMPENSATION

We will give you a $20 gift card for completing the survey and focus group, to thank you for your time and effort.

CONFIDENTIALITY

You gave us your name and phone number when you expressed interest in the study. However, we assigned a study code to you and we will not ask you to report any personal information (such as your full name, address, full date of birth) in front of other study participants. Your personal information is stored in a locked filing cabinet and only study staff has access to it.. We will not tell anyone that you participated in the study. However, your participation is not entirely anonymous because the other focus group participants will know that you took part. Even if you do not give your name during the focus group, it is possible that you will be identified by other focus group participants, especially if the community you come from is small. We will ask focus group participants not to tell others who attended the focus group or what was discussed. However, we cannot promise that all participants will follow our request so the information you share during the focus group is not entirely confidential. Otherwise, we will fully protect your anonymity and the confidentiality of the information you give us.

We will store information you provide us that is recorded on paper and on tape recordings in locked filing cabinets. We will store information that is recorded electronically in password protected files. We will use a study code instead of your name on all study files. We will destroy all information seven years after the study is finished.

None of the information you provide us will be shared with anyone outside of the research team, unless the law requires us to do this. Your individual answers will not be shared with doctors, treatment providers, police or other criminal justice people. However, in some circumstances we legally have to report information to the authorities, for example: 1) if you tell me that you are going to harm yourself or others, 2) if there is a court warrant requiring access to the information, 3) if we suspect child abuse of anyone under 16 years old who may need protection from physical, sexual or emotional abuse, neglect, or risk of harm.

Your research records might also be accessed so the study can be monitored or audited by: 1) a member of the Quality Assurance Team as part of CAMH’s Research Services Quality Assurance Program, 2) on behalf of the Research Ethics Board as part of continuing review of the research. In both cases, confidentiality will be maintained as per CAMH policies and to the extent permitted by law.

We might publish the results of this study in a journal article or present them at a scientific conference. We will never refer to you by your name or identify you in any other way. We might quote you, but we will make sure that you cannot reasonably be identified from the quote.

Participant Initials: _____

YOUR RIGHTS

You have the right at any time to ask questions about the study and discuss your concerns with the research team. Your participation is completely voluntary. You may skip any question or part of the study. You may withdraw from the survey and/or focus group at any time without penalty. If you do withdraw from the study, and you would like us to withdraw your information, we will do so to the best of our abilities. We reserve the right to end your participation in the study at any time. Results of this study will only be used for scientific purposes. If we use study results in future research, your rights will be protected just like they are now. By participating in this study, you do not give up any of your legal rights or free the researchers and the Centre for Addiction and Mental Health of their legal and professional responsibilities.

CONTACT INFORMATION

This study is being carried out by Dr. Pamela Sabioni (Principal Investigator), Dr. Joanna Henderson and Dr. Jürgen Rehm at the Centre for Addiction and Mental Health (CAMH). This research is funded by the Canadian Institutes of Health Research (CIHR).

If you would like more information about this study or if you have any questions or concerns, you may contact:

| Principal Investigator |  |
| --- | --- |
| Dr. Pamela Sabioni |  |
| Centre for Addiction and Mental Health |  |
| 1-800-535-8501 ext. 33094 |  |

To discuss your rights as a participant you can contact:

| Research Ethics Board Chair |
| --- |
| Dr. Robert Levitan |
| Centre for Addiction and Mental Health |
| 1-800-535-8501 ext. 34020 |

Participant Initials: _____

**Survey and Focus Group Oral Consent Documentation**

**NODUS Needs Assessment**

**Northern Ontario Drug Use and Services Needs Assessment**

*Instruction to researcher: Please check each item to document that it has been covered during the consent process.*

The participant has:

- had ample time to consider participation in the study
- been able to ask all his/her questions to the study staff
- read the information letter entirely or it has been read to him/her
- been offered a copy of the information letter and consent form to keep

The participant has indicated that he/she understands:

- the study procedure, including technical language used to describe the study
- that he/she can contact study staff with additional questions about the study
- that all information will be kept confidential, within the limits outlined
- that he/she may withdraw from this study at any time without penalty or loss of benefits
- that his/her decision whether to participate will not change any of his/her legal rights or health care entitlements
- The participant has voluntarily consented to participate in this study

|  | Researcher’s name |  | Time |
| --- | --- | --- | --- |
|  | Researcher’s signature |  | Date |

Participant Initials: ____________
