# Supplementary material for: Assessing service and treatment needs of young people who use illicit and non-medical prescription drugs living in Northern Ontario, Canada
Source: F1000Res. 2019 Oct 28;7:1644. Originally published 2018 Oct 16. [Version 2] doi: 10.12688/f1000research.16464.2 (PMC7025771; doi:10.12688/f1000research.16464.2)
Supplement: Supplementary file 2 [file f1000research-7-23219-s0001.tgz › 26391d49-5c1f-4a9f-b399-8fda5a5b07e8_NODUS.Protocol.F1000Research.Youth.FocusGroupGuide.docx]

## ****Focus Group Guide****

## ****Instructions****

a) In today’s discussion, we would like for everyone to participate and share their thoughts and opinions.

b) There is no right or wrong answer. We also do not want you to feel judged because of your drug use. We are here so we can learn how to best to respond to the needs of youth who use drugs.

c) Today’s discussion will be audio taped to avoid misinterpretation and to ensure all information is captured. Only the study investigators and study staff assisting with this research study will have access to the tapes.

d) Everything that is said in our discussion today is treated as confidential. Please do not tell anyone who was here or what was talked about today.

e) In our discussion, you are not required to answer questions based on your own experiences. If you are more comfortable doing so, you can talk about young people you know who use drugs rather than specifically about yourself.

f) Please be careful about discussing drug use in such a way that it might trigger someone to want to use drugs. For example, we do not want to graphically discuss the methods of using drugs.

g) Please let us know if you feel triggered and/or need clinical support.

h) We ask that only one person speaks at a time and everyone is being respectful when an individual is speaking.

i) Just a reminder, we will only be focusing on any drug except alcohol or marijuana.

## ****Focus Group Questions****

### Substance use and associated harms

1. Other than alcohol or marijuana, what drugs do you or your friends use?
   - Which drugs do you most often use?
   - How do you or your friends administer the drugs that you just listed? Ex: do you snort them? Inject? Oral?
2. Where do you or your friends mostly use drugs?
   - Why do you go to those places to use drugs?
3. Do you think using drugs has affected your health or the health of someone else you know?
   - How have the drugs you used affected your health?
4. What aspects of drug use do you think harms your health or puts your health at risk?
   - Ex: think about your environment or the way in you administer the drug or how much you administer
5. Do you do something risky after using drugs?
   - Ex: When you’re intoxicated with the drug, do you do something that you normally would not do?

### Service use and barriers

1. What services or treatments for drug use are available in your community?
   - Do you know where you or your friend could go if you want to access services for drug use?
2. Do you go to these services or treatment centers often? Why? Which ones do you most often visit?
   - Why don’t you go to these services often?
3. What services or treatments do you find to be the most helpful/beneficial?
   - Why are those services beneficial?
   - Have the treatments and services that you have accessed worked for you or do you think were appropriate for your specific case?
4. Is there anything that you don’t like about the existing services or treatment centers you have accessed?
   - Would like to change or improve anything about these existing services or treatment centers?
5. What are some barriers or obstacles in using or accessing these services or treatment centers?

**Overdose**

1. If you or someone you know is overdosing, do you know any services that can help you to prevent or manage an overdose?
2. What would you do or have you done if you saw someone overdose?

### Service needs

1. Are there any services that you need/want that you cannot find or access?

### Other comments

1. Is there anything else you would like to talk about regarding drug use among youth?
2. Is there anything you wanted to say that you didn’t get a chance to talk about before?
