# Supplementary material for: Assessing service and treatment needs of young people who use illicit and non-medical prescription drugs living in Northern Ontario, Canada
Source: F1000Res. 2019 Oct 28;7:1644. Originally published 2018 Oct 16. [Version 2] doi: 10.12688/f1000research.16464.2 (PMC7025771; doi:10.12688/f1000research.16464.2)
Supplement: Supplementary file 3 [file f1000research-7-23219-s0002.tgz › 361d105f-8e8f-48b9-bc09-b54002ff7ff9_NODUS.Protocol.F1000Research.Youth.Questionnaire.docx]

**Participation Code:** NODUSY______ **Date:**_____________

**SERVICE AVAILABILITY**

Q1. Are these SERVICES AVAILABLE in your COMMUNITY?

| Services | Yes | No | I don’t know | I prefer not to answer |
| --- | --- | --- | --- | --- |
| Social services (welfare, disability, etc.) |  |  |  |  |
| Drop-in center |  |  |  |  |
| Food bank |  |  |  |  |
| Shelter |  |  |  |  |
| Doctor's office or walk-in clinic |  |  |  |  |
| Emergency room |  |  |  |  |
| Psychiatric hospital |  |  |  |  |
| Detoxification |  |  |  |  |
| Residential addiction treatment program (inpatient) |  |  |  |  |
| Community addiction treatment program (outpatient) |  |  |  |  |
| Counselling, support group |  |  |  |  |
| Assisted housing (halfway house, transition house, group house) |  |  |  |  |
| Peer support worker |  |  |  |  |
| Caseworker (CAS, Probation, etc.) |  |  |  |  |
| Sponsor (AA, NA, etc.) |  |  |  |  |
| Needle exchange, crack kit distribution |  |  |  |  |
| Methadone/Suboxone/Buprenorphine maintenance program |  |  |  |  |
| Naloxone distribution program |  |  |  |  |
| Street outreach or mobile program |  |  |  |  |

Q1(a). Please list any **SERVICES** that are **AVAILABLE** in your **COMMUNITY** that were not listed in **QUESTION 1**:

**SERVICE USE**

Q2. Have you **USED** the following **SERVICES**?

| Services | I have used this service | | | | | |
| --- | --- | --- | --- | --- | --- | --- |
|  | **More than a year ago** | **Within the last year** | **Within the last month** | **Never** | **I don’t know** | **I prefer not to answer** |
| Social services (welfare, disability, etc.) |  |  |  |  |  |  |
| Drop-in center |  |  |  |  |  |  |
| Food bank |  |  |  |  |  |  |
| Shelter |  |  |  |  |  |  |
| Doctor's office or walk-in clinic |  |  |  |  |  |  |
| Emergency room |  |  |  |  |  |  |
| Psychiatric hospital |  |  |  |  |  |  |
| Detoxification |  |  |  |  |  |  |
| Residential addiction treatment program (inpatient) |  |  |  |  |  |  |
| Community addiction treatment program (outpatient) |  |  |  |  |  |  |
| Counselling, support group |  |  |  |  |  |  |
| Assisted housing (halfway house, transition house, group house) |  |  |  |  |  |  |
| Peer support worker |  |  |  |  |  |  |
| Caseworker (CAS, Probation, etc.) |  |  |  |  |  |  |
| Sponsor (AA, NA, etc.) |  |  |  |  |  |  |
| Needle exchange, crack kit distribution |  |  |  |  |  |  |
| Methadone/Suboxone/Buprenorphine maintenance program |  |  |  |  |  |  |
| Naloxone distribution program |  |  |  |  |  |  |
| Street outreach or mobile program |  |  |  |  |  |  |

Q3. Have you **USED** any **SERVICES** for drug use **OUTSIDE** of your **COMMUNITY**?

- Yes. Please list what services you used and where. ____________________________________­­­­

_____________________________________________________________________________

- No
- I don’t know
- I prefer not to answer

Q4: How **HELPFUL** were the **SERVICES** you **USED**?

| Services | Did not Use | Extremely helpful | Very helpful | Somewhat helpful | Slightly helpful | Not at all helpful | I don’t know | I prefer not to answer |
| --- | --- | --- | --- | --- | --- | --- | --- | --- |
| Social services (welfare, disability, etc.) |  |  |  |  |  |  |  |  |
| Drop-in center |  |  |  |  |  |  |  |  |
| Food bank |  |  |  |  |  |  |  |  |
| Shelter |  |  |  |  |  |  |  |  |
| Doctor's office or walk-in clinic |  |  |  |  |  |  |  |  |
| Emergency room |  |  |  |  |  |  |  |  |
| Psychiatric hospital |  |  |  |  |  |  |  |  |
| Detoxification |  |  |  |  |  |  |  |  |
| Residential addiction treatment program (inpatient) |  |  |  |  |  |  |  |  |
| Community addiction treatment program (outpatient) |  |  |  |  |  |  |  |  |
| Counselling, support group |  |  |  |  |  |  |  |  |
| Assisted housing (halfway house, transition house, group house) |  |  |  |  |  |  |  |  |
| Peer support worker |  |  |  |  |  |  |  |  |
| Caseworker (CAS, Probation, etc.) |  |  |  |  |  |  |  |  |
| Sponsor (AA, NA, etc.) |  |  |  |  |  |  |  |  |
| Needle exchange, crack kit distribution |  |  |  |  |  |  |  |  |
| Methadone/Suboxone/  Buprenorphine maintenance program |  |  |  |  |  |  |  |  |
| Naloxone distribution program |  |  |  |  |  |  |  |  |
| Street outreach or mobile program |  |  |  |  |  |  |  |  |
| Other (please specify): |  |  |  |  |  |  |  |  |
|  |  |  |  |  |  |  |  |  |

SERVICE NEEDS

Q5. Which of these services do you **NEED** or **WANT** to use?

| Services | I need/want to use this service | I need/want to use this service but can’t access it | I need/want to use this service but it isn’t available | I do not need/want this service | I don’t know | I prefer not to answer |
| --- | --- | --- | --- | --- | --- | --- |
| Social services (welfare, disability,  etc.) |  |  |  |  |  |  |
| Drop-in center |  |  |  |  |  |  |
| Food bank |  |  |  |  |  |  |
| Shelter |  |  |  |  |  |  |
| Doctor's office or walk-in clinic |  |  |  |  |  |  |
| Emergency room |  |  |  |  |  |  |
| Psychiatric hospital |  |  |  |  |  |  |
| Detoxification |  |  |  |  |  |  |
| Residential addiction treatment program (inpatient) |  |  |  |  |  |  |
| Community addiction treatment program (outpatient) |  |  |  |  |  |  |
| Counselling, support group |  |  |  |  |  |  |
| Assisted housing (halfway house, transition house, group house) |  |  |  |  |  |  |
| Peer support worker |  |  |  |  |  |  |
| Caseworker (CAS, Probation, etc.) |  |  |  |  |  |  |
| Sponsor (AA, NA, etc.) |  |  |  |  |  |  |
| Needle exchange, crack kit distribution |  |  |  |  |  |  |
| Methadone/Suboxone/Buprenorphine maintenance program |  |  |  |  |  |  |
| Naloxone distribution program |  |  |  |  |  |  |
| Street outreach or mobile program |  |  |  |  |  |  |
| Other (please specify): |  |  |  |  |  |  |

Q6. What **SERVICES** related to **DRUG USE** do you feel are **MISSING** in your **COMMUNITY**?

­­­­­­­­­­­­­­­­­­­­­­­­­­­­­­­­­­­­­­­­­­­­­­­­­­­­­­­­­­­­­­­­­­­­­­­­­­­­­­­­­

Q7. What are the **MAIN BARRIERS** in **ACCESSING SERVICES** for **DRUG USE** in your **COMMUNITY**?

­­­­­­­­­­­­­­­­­­­­­­­­­­­­­­­­­­­­­­­­­­­­­­­­­­­­­­­­­­­­­­­­­­­­­­­­­­­­­­­­­

­­­­­­­­­­­­­­­­­­­­­­­­­­­­­­­­­­­­­­­­­­­­­­­­­­­­­­­­­­­­­­­­­­­­­­­­­­­­­­­­­

**SOCIO DEMOGRAPHICS**

Q8. How **OLD** are you? ______________

Q9. What is your **GENDER**? (C*heck as many as apply*)

- Man
- Woman
- Transgender
- Non-binary
- I don't know
- I prefer not to answer
- Other (*please specify*): __________________________________

Q10. Which **BACKGROUND(S)** do you **SELF-IDENTIFY** with? (*Check as many as apply*)

- White
- Black
- Asian
- Latin American
- Middle Eastern
- Aboriginal
- I don't know
- I prefer not to answer
- Other (*please specify*): __________________________________

Q11. Where do you **LIVE**? (C*heck as many as apply*)

- My own place (house/condo/apartment)
- Living with parents/guardians
- Living with friends
- Living with relatives
- Shelter
- Assisted housing
- I don't know
- I prefer not to answer
- Other (*please specify*): __________________________________

Q12. What is your **HIGHEST LEVEL OF EDUCATION**?

- Did not finish elementary school
- Graduated from elementary school
- Did not finish high school
- Graduated from high school
- Graduated from university/college
- I don't know
- I prefer not to answer
- Attended university/college but did not complete degree
- Other (*please specify*): __________________________________

Q13. In the **LAST MONTH**, what type of **EDUCATION** or **EMPLOYMENT ACTIVITIES** have you been **INVOLVED** in? (*Check as many as apply*)

- High school student
- Post-secondary school student (college, university)
- Working
- Training for a job/trade
- Providing family care
- I don't know
- I prefer not to answer
- Other (*please specify*): __________________________________

**INCOME**

Q14. In the **LAST MONTH**, what were your **SOURCES OF MONEY**? (*Check as many as apply*)

- Legal work (full-time, part-time, temporary, casual)
- Government assistance (e.g. welfare, disability)
- Parents
- Relatives
- Friends
- Soliciting
- Illegal activities
- I don't know
- I prefer not to answer
- Other (*please specify*): ________________________________________

Q15. In the **LAST MONTH, HOW MUCH MONEY** did you make from **ALL THE DIFFERENT SOURCES** you checked in the previous question?

- $0-249
- $250-499
- $500-749
- $750-999
- $1000-1999
- $2000 or more
- I don't know
- I prefer not to answer

HEALTH

Q16. How would you rate your overall PHYSICAL HEALTH status in the LAST MONTH? In general, would you say your PHYSICAL HEALTH is:

- Excellent
- Very Good
- Good
- Fair
- Poor
- I don't know
- I prefer not to answer

Q17. How would rate your overall **MENTAL/EMOTIONAL HEALTH** status in the **LAST MONTH**? In general, would you say your **MENTAL/EMOTIONAL HEALTH** is:

- Excellent
- Very Good
- Good
- Fair
- Poor
- I don't know
- I prefer not to answer

Q18. Have you **EVER** been **DIAGNOSED** with any of the following **MENTAL HEALTH ISSUES**?

| Mental health Issues | Yes | No | I don't know | I prefer not to answer |
| --- | --- | --- | --- | --- |
| Depression |  |  |  |  |
| Anxiety disorders |  |  |  |  |
| Substance use disorders |  |  |  |  |
| Schizophrenia or psychosis |  |  |  |  |
| Personality disorders |  |  |  |  |
| Other mental health issues (*please specify*): | | | | |

DRUG USE

Q19. Which of the following **DRUGS** did you **USE** on **MORE THAN 10 DAYS** in the **LAST MONTH**? *(Please exclude medical prescriptions from a doctor)*

| Drug Types | Used on MORE THAN 10 DAYS | Did NOT use on MORE THAN 10 DAYS | I don’t know | I prefer not to answer |
| --- | --- | --- | --- | --- |
| Alcohol |  |  |  |  |
| Tobacco |  |  |  |  |
| Cannabis (Marijuana, Hash) |  |  |  |  |
| Cocaine |  |  |  |  |
| Crack-Cocaine |  |  |  |  |
| Heroin |  |  |  |  |
| Gambutrol |  |  |  |  |
| Ecstasy (MDMA) |  |  |  |  |
| Hallucinogens (mushrooms, acid, PCP, ketamine) |  |  |  |  |
| Glue or other solvents (gasoline, butane, aerosols, etc.) |  |  |  |  |
| Speed/Amphetamines |  |  |  |  |
| Methamphetamine/Crystal Meth |  |  |  |  |
| Codeine (T3s, T4s) |  |  |  |  |
| Morphine (MS Contin, Statex) |  |  |  |  |
| Meperidine (Demerol) |  |  |  |  |
| Hydrocodone (Tussionex, Dalmacol) |  |  |  |  |
| Oxycodone (OxyNeo, Oxycocet, Percocet, Percodan) |  |  |  |  |
| Hydromorphone (Dilaudid) |  |  |  |  |
| Fentanyl (Duragesic) |  |  |  |  |
| Methadone |  |  |  |  |
| Buprenorphine (Suboxone) |  |  |  |  |
| Other prescription opioids (oxymorphone, tramadol, pentazocine) |  |  |  |  |
| Benzodiazepines (Xanax, Valium, Ativan) |  |  |  |  |
| Barbiturates (Nembutal, Luminal) |  |  |  |  |
| ADHD medication (Ritalin, Concerta, Adderall) |  |  |  |  |
| Anti-depressants (Prozac, Wellbutrin, Elavil) |  |  |  |  |
| Other (Please Specify): |  |  |  |  |

Q20. Have you **EVER INJECTED DRUGS**?

- Yes. What drugs have you injected? _________________________________________________
- No
- I don't know
- I prefer not to answer

Q21. Have you ever EXPERIENCED an OVERDOSE?

- Yes. Please list WHAT DRUGS you have OVERDOSED on and HOW MANY TIMES you have OVERDOSED. ___________________________________________________________________
- No
- I don't know
- I prefer not to answer

Q22. Which of the following **PROBLEMS** have **YOU EXPERIENCED** because of your **DRUG USE**? (*Check as many as apply*)

- Health problems. Please specify the **HEALTH PROBLEMS** you have.________________________

______________________________________________________________________________

- School problems (e.g. you miss school days because of your drug use, you missed deadlines, have problems focusing, etc.)
- Work problems (e.g. you missed work days because of your drug use, have problems focusing)
- Social problems (e.g. you have problems in your relationship with family or friends, you miss important events, etc.)
- Legal problems (e.g. you have been involved in criminal activities, have been arrested, etc.)
- Financial problems
- I have not experienced any problems
- I don’t know
- I prefer not to answer

**CONGRATULATIONS!**

You have come to the end of the questionnaire. Thank you for your participation!

Please let the research staff know that you are done.
