# Supplementary material for: Assessing service and treatment needs of young people who use illicit and non-medical prescription drugs living in Northern Ontario, Canada
Source: F1000Res. 2019 Oct 28;7:1644. Originally published 2018 Oct 16. [Version 2] doi: 10.12688/f1000research.16464.2 (PMC7025771; doi:10.12688/f1000research.16464.2)
Supplement: Supplementary file 4 [file f1000research-7-23219-s0003.tgz › 4874dc9b-e1d0-4eb6-b768-67019d7e51d3_NODUS.Protocol.F1000Research.KeyInformant.InterviewGuide.docx]

## ****Key Informant Interview Guide****

## ****Consent****

Prior to interview, participant will read the information letter and sign the consent form. For phone interviews, participant will send the signed consent form before the interview begins.

## ****Questions****

### Key Informant’s role

1. What is your current job?
2. What is your background and training?
3. What parts of your job are relevant to youth who use drugs?
4. What age group do you typically work with?
5. How long have you been in the role?
6. How long have you been working in [community name]?

### Substance use and associated harms

1. Can you describe to me what drug use among youth and young adults typically looks like within your community?
2. What are the main drugs that are used by youth in your community?
3. Where do youth in your community mostly use drugs?
4. What aspects of drug use do you think harms or puts the health of youth at risk? (For example, think about the environment or the way in which they administer the drug.)

### Service use and barriers

1. What services or treatment centers for drug use (e.g., harm reduction, treatment, detox) are available in your community?
2. Which of the services or treatment centers do youth use most often?
3. Which services or treatment centers are the most helpful/ beneficial in your community?
4. What do you think could be improved about existing services? How?
5. What are some barriers or obstacles to using or accessing existing services or treatment centers?

### Service needs

1. What services or treatment centers do you think would be beneficial/ helpful for you drug users to have in your community that you cannot find or access now?
2. What are the barriers to implementing new services in your community?

### Other comments

1. Is there anything else you think we should know that we haven’t covered yet?

## ****Concluding remarks****

1. Is there anyone else it is important we talk to in your community?
