# Supplementary material for: Assessing service and treatment needs of young people who use illicit and non-medical prescription drugs living in Northern Ontario, Canada
Source: F1000Res. 2019 Oct 28;7:1644. Originally published 2018 Oct 16. [Version 2] doi: 10.12688/f1000research.16464.2 (PMC7025771; doi:10.12688/f1000research.16464.2)
Supplement: Peer review history

# Peer review: Reviewer response for version 2

**DOI:** 10.5256/f1000research.23219.r55778

**Published:** 2020-02-14

**Authors:** Tess K. Drazdowski

**Affiliations:** 1 Oregon Social Learning Center, Eugene, OR, USA

The authors have sufficiently addressed the original concerns raised.

I confirm that I have read this submission and believe that I have an appropriate level of expertise to confirm that it is of an acceptable scientific standard.

# Peer review: Reviewer response for version 2

**DOI:** 10.5256/f1000research.23219.r55779

**Published:** 2019-11-25

**Authors:** Marilyn Clark

**Affiliations:** 1 University of Malta, Msida, Malta

I confirm that I have read this submission and believe that I have an appropriate level of expertise to confirm that it is of an acceptable scientific standard.

# Peer review: Reviewer response for version 1

**DOI:** 10.5256/f1000research.17996.r43269

**Published:** 2019-07-10

**Authors:** Tess K. Drazdowski

**Affiliations:** 1 Oregon Social Learning Center, Eugene, OR, USA

This article presents a study protocol for a needs assessment research project aimed at determining the substance use treatment needs of youth and young adults who currently use illicit substances (including the non-medical use of prescription drugs) in Northern Ontario. Overall, the study protocol is set to examine an important public health problem in areas that are typically under served (remote and rural communities). An innovative feature of the protocol includes a mobile research lab. Other strengths are the mixed method design, plans to interview service providers and community organizers as well as the youth and young adults, and to conduct 1-on-1 interviews with youth and adults if not enough are available for a focus group.

 Some points of consideration that may improve the study protocol are listed below:

1. First, given that the areas of interest are likely not densely populated the inclusion criteria may be too stringent to get a large enough sample of youth and young adults to be representative of the substance using population in these areas. Specially, needing to have misused substances for at least 10 days in the last month (especially for prescription drug misuse where only 5-12% report misuse in the past year) seems as though it may eliminate a lot of individuals who would otherwise qualify if the threshold was lower. Information for why these thresholds were chosen would be helpful.
2. Rationale for why there is no compensation for service providers for their participation is recommended.
3. The authors mention solvent abuse in the introduction as a problem, however it is not clear if that was included in the screening of participants. It is noted that it was included in the data collection.

I confirm that I have read this submission and believe that I have an appropriate level of expertise to confirm that it is of an acceptable scientific standard, however I have significant reservations, as outlined above.

# Response

**Published:** 2019-10-21

**Authors:** Thepikaa Varatharajan

**Affiliations:** Centre for Addiction and Mental Health, Toronto, Canada

Thank you for reviewing our article. Please see our responses below.

***This article presents a study protocol for a needs assessment research project aimed at determining the substance use treatment needs of youth and young adults who currently use illicit substances (including the non-medical use of prescription drugs) in Northern Ontario. Overall, the study protocol is set to examine an important public health problem in areas that are typically underserved (remote and rural communities). An innovative feature of the protocol includes a mobile research lab. Other strengths are the mixed method design, plans to interview service providers and community organizers as well as the youth and young adults, and to conduct 1-on-1 interviews with youth and adults if not enough are available for a focus group. Some points of consideration that may improve the study protocol are listed below.***

Thank you for your comments.

***First, given that the areas of interest are likely not densely populated the inclusion criteria may be too stringent to get a large enough sample of youth and young adults to be representative of the substance using population in these areas. Specially, needing to have misused substances for at least 10 days in the last month (especially for prescription drug misuse where only 5-12% report misuse in the past year) seems as though it may eliminate a lot of individuals who would otherwise qualify if the threshold was lower. Information for why these thresholds were chosen would be helpful.***

The criteria for participants to have misused substances for at least 10 days in the last month allows us to capture youth who are frequently misusing drugs (i.e., on daily/more than weekly basis).  Youth who are misusing drugs regularly are more likely to seek services and treatment centres for their drug use than youth who are experimenting with drugs.

***Rationale for why there is no compensation for service providers for their participation is recommended.***

We saw service providers as key informants. Key informants in this study either held a formal position in the community that allowed them to directly serve or interact with young people who use drugs (e.g., social worker, addictions counsellor), or held a position in which they influence services, decisions or policies that affect young people who use drugs (e.g., police chief, town councillor).

These individuals were not only recruited to participate in the study, but also played a key role in helping us recruit participants and planning study logistics, i.e. they were part of the study team. We considered paying an honorarium, and informally asked about this, and received advice that this was not seen appropriate, since our key informants were participating in a professional capacity and were keen to help in any way they can. Service providers did, however, show a lot of interest in contributing to and/or receiving a community specific report with study findings.

***The authors mention solvent abuse in the introduction as a problem, however it is not clear if that was included in the screening of participants. It is noted that it was included in the data collection.***

During the screening process participants were asked if they had used any illicit or non-medical prescription drugs which includes inhalants such as gases, solvents and aerosol sprays.

# Peer review: Reviewer response for version 1

**DOI:** 10.5256/f1000research.17996.r44607

**Published:** 2019-03-14

**Authors:** Marilyn Clark

**Affiliations:** 1 University of Malta, Msida, Malta

The study protocol provides a clear and realistic research design.

 The protocol should clearly indicate whether or not parental consent is required by law (in Ontario) to be sought for the participation of minors in addition to consent from the minors themselves. Some indication of what the legal requirements in this regard are in Canada would be useful. Provincial legislation and other applicable legal and regulatory requirements related to legal age of consent should be included in the protocol.

 The ethical principle of respect for persons recommends that parental permission and minor consent function together to protect the child. The researchers' decision to not seek parental permission because it would disclose the behaviour of the minors to their parents is understandable but it needs to be pitted against the possible risks for the minor and the rights of the parents. 

 Acceptance recommended if the issue of parental consent is addressed in more detail with reference to the attendant legislation.

I confirm that I have read this submission and believe that I have an appropriate level of expertise to confirm that it is of an acceptable scientific standard, however I have significant reservations, as outlined above.

## References

[1] : Parental permission and child assent in research on children. *Yale J Biol Med*.2013;86(3) :291-301 24058304PMC3767214

# Response

**Published:** 2019-04-27

**Authors:** Thepikaa Varatharajan

**Affiliations:** Centre for Addiction and Mental Health, Toronto, Canada

Thank you for reviewing our protocol and providing your comments.

 According to Health Canada and the Public Health Agency of Canada's Research Ethics Board (REB), in Ontario parental/guardian consent is required for children under the age of 16. However, youth assessed and determined as “mature minors” may also provide consent. A “mature minor” is a person under 16 years of age who can demonstrate adequate understanding and decision-making capacity (i.e. being able to fully understand the nature, risks and benefits of a proposed study).

 In line with legal requirements, we did not seek parental consent for individuals aged 14-16. This research approach was discussed exclusively before REB approval (Centre for Addiction and Mental Health’s REB protocol reference #133/2015):

*“Given the low risk nature of the study, youth 14-16 years old will be eligible for the study, but only if they have the capacity to consent (i.e. able to understand the purpose of the research, what is involved and potential consequences). Study staff will be trained by clinical staff to judge capacity by gauging whether the potential participant has an understanding of the study and the consequences of participation, for example, by asking the participant to summarize the study and to answer questions about the study during the eligibility screener.* *Youth who are not capable of providing informed consent are not eligible for the study.”*

 In addition, we made sure that no false decisions were taken with respect to the ability to give informed consent. The following procedures were introduced (cited from our protocol):

*“To ensure that all participants (including those who are under 16 years of age or may not be capable of consent) understand the study and the possible consequences of participation, study staff will review the consent form with the participants and clarify any questions the participants may have. Research staff working with participants will be trained by a CAMH clinical physician to judge capacity by assessing acute intoxication and other relevant impairments that may interfere with the participant’s ability to consent.”*
